# Electrical Properties of Polyetherimide-Based Nanocomposites Filled with Reduced Graphene Oxide and Graphene Oxide-Barium Titanate-Based Hybrid Nanoparticles

**DOI:** 10.3390/polym14204266

**Published:** 2022-10-11

**Authors:** Quimberly Cuenca-Bracamonte, Mehrdad Yazdani-Pedram, Héctor Aguilar-Bolados

**Affiliations:** 1 Facultad de Ciencias Químicas y Farmacéuticas, Universidad de Chile, Olivos 1007, Santiago 8380544, Chile; 2 Departamento de Polímeros, Facultad de Ciencias Químicas, Universidad de Concepción, Concepción 3349001, Chile

**Keywords:** polyetherimide nanocomposite, hybrid graphene materials, reduced graphene oxide

## Abstract

The electrical properties of nanocomposites based on polyetherimide (PEI) filled with reduced graphene oxide (rGO) and a graphene oxide hybrid material obtained from graphene oxide grafted with poly(monomethyl itaconate) (PMMI) modified with barium titanate nanoparticles (BTN) getting (GO-g-PMMI/BTN) were studied. The results indicated that the nanocomposite filled with GO-g-PMMI/BTN had almost the same electrical conductivity as PEI (1 × 10^−11^ S/cm). However, the nanocomposite containing 10 wt.% rGO and 10 wt.% GO-g-PMMI/BTN as fillers showed an electrical conductivity in the order of 1 × 10^−7^ S/cm. This electrical conductivity is higher than that obtained for nanocomposites filled with 10% rGO (1 × 10^−8^ S/cm). The combination of rGO and GO-g-PMMI/BTN as filler materials generates a synergistic effect within the polymeric matrix of the nanocomposite favoring the increase in the electrical conductivity of the system.

## 1. Introduction

Graphene materials, such as graphene oxide (GO) and reduced graphene oxide (rGO), have been widely studied due to their ease of obtainment and excellent properties. For instance, rGO presents excellent electrical properties, while GO has oxygen moieties that impart reactivity to be functionalized using different compounds [1,2,3]. Moreover, the use of graphene oxide and reduced graphene oxide as fillers in polymer nanocomposites to improve their thermal, electrical, and mechanical properties has increased since the advent of graphene materials. This has generated interest in scientific and technological fields, as well as an important advance in different areas [4,5].

The GO corresponds to oxidized graphene sheets, which present oxygenated functional groups such as hydroxyl, epoxide, carboxylic acid, ketones, and cyclic esters [6,7]. The presence of these functional groups favors the exfoliation of graphene layers by using thermal reduction as a top-down method. On the other hand, GO is a less conductive material than graphite since the carbon atoms bonded to oxygen functional groups present sp^3^-hybridization, which disrupts the continuity of the long-range sp^2^ conjugated system, affecting the charge transport lattice characteristics [8].

The GO can be reduced using different methods to obtain the rGO. One of these methods is thermal reduction, which consists of heating GO above 600 °C, which results in a fast reduction and rapid exfoliation of graphene oxide layers. The exfoliation is the result of the thermal decomposition of oxygenated functional groups present in GO, which yield gaseous molecules, such as CO_2_, CO, and H_2_O [6,7].

As is known, there are a wide variety of graphene materials, where among them polymer-grafted graphene oxide is highlighted because the polymer assists the compatibility of the graphene with the environment where it is dispersed [9]. Polymer-grafted graphene oxides can be obtained by the grafting-from, as well as the grafting-to methods [10]. In this regard, previously our group reported the obtaining of a grafted-from graphene oxide, where the grafting polymer was poly (monomethyl itaconate), and the route to obtain consisted of atom transfer radical polymerization [11]. It is important to notice that the monomer, monomethyl itaconate, corresponds to a derivative of the itaconic acid. Itaconic acid is obtained by a fermentation process of renewable sources [12].

On the other hand, graphene materials, such as graphene oxide and its derivatives, are used as fillers in polymers to improve the performance of properties such as electrical, mechanical, and barrier properties [13]. One of the challenges in this matter is the filler dispersion. In this respect, poly (monomethyl itaconate) grafted onto graphene oxide can be an alternative to assist the dispersion of the filler in a polymeric matrix.

On the other hand, barium titanate (BT) is a perovskite-type oxide considered a ferroelectric material with high relative permittivity [14]. Barium titanate nanoparticles (BTN) are generally used for capacitor design or as filler in dielectric elastomers to improve their dielectric permittivity [15,16]. Some studies have shown improvements in dielectric, stress-strain mechanical, and energy storage properties as results of the use of barium titanate nanoparticles for obtaining polymer-based nanocomposites [17]. In particular, an improvement in the dielectric breakdown strength was observed, which is attributed to the increase in the degree of charge dispersion and the mitigation of the local electric field concentration promoted by GO. The introduction of ceramic fillers with BTNs into a polymer matrix is an effective procedure to obtain dielectric nanocomposites with high energy storage density [18]. Other authors have reported that by using BTN and GO, composites with high dielectric strength can be obtained [19].

Although studies have been carried out regarding GO and BTN-filled nanocomposites, these have been prepared by physically mixing the components of nanocomposites. Hence, it is possible to think about the modification of GO with BTN, where BTN is covalently linked to GO.

**Figure 1:**
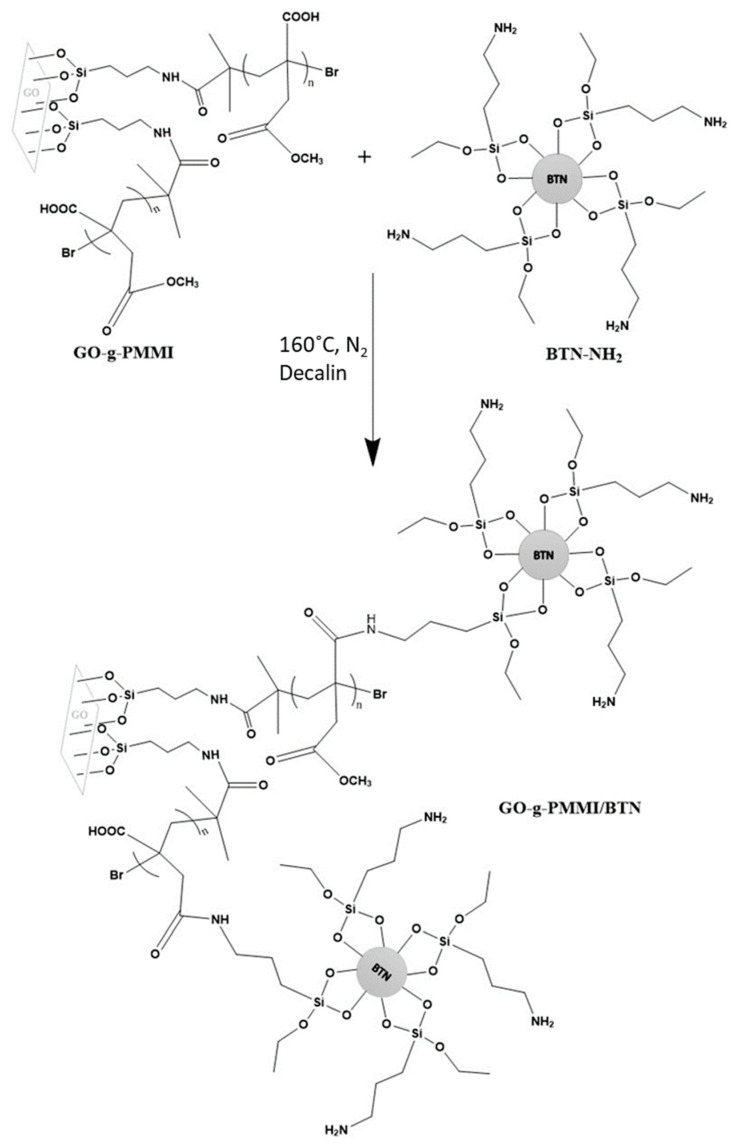
Proposed reaction scheme between silanized barium titanate nanoparticles (BTN-NH2) and GO-g-PMMI to yield hybrid GO-g-PMMI/BTN.

To obtain hybrid materials based on functionalized graphene oxide and barium titanate nanoparticles, it is necessary to modify the structure of both materials. Therefore, a novel approach to synthesize these hybrid materials is to use polymer-grafted graphene oxide and functionalized barium titanate nanoparticles that, by a reaction yield, graphene oxide covalently bonds to the BTNs. In this regard, the poly (monomethyl itaconate)-grafted-graphene oxide can react with barium titanate functionalized with amine groups by nucleophilic substitution reaction. The amine-functionalized barium titanate is prepared by subsequent reactions of hydroxylation and silanization by using 3-aminopropyltriethoxysilane [20]. We hypothesize that the carboxylic acid and ester side groups of poly(monomethyl itaconate) are susceptible to the nucleophilic attack of amine groups, promoting the formation of covalent bonds that links graphene oxide sheets with barium titanate nanoparticles (Figure 1). In this work, the hybrid material prepared by this approach is designated as GO-g-PMMI/BTN and its use as filler for the preparation of nanocomposites based on polyether imide (PEI) was investigated. PEI corresponds to a high-performance thermoplastic polymer with excellent mechanical and thermal properties. The effect of the use of the hybrid graphene materials in combination with reduced graphene oxide was also evaluated. The aim of this work is to evaluate the electrical properties, such as electrical conductivity and dielectric permittivity, of nanocomposites based on polyetherimide and hybrid graphene and/or reduced graphene oxide prepared by a solvent casting method.

## 2. Materials and Methods

### 2.1. Materials

Graphite (+100 mesh (≥75% min)), polyetherimide (melt index 18 g/10 min (337 °C/6.6 kG)), and barium titanate (IV) nanopowder (cubic) 50 nm 99.9% were supplied by Aldrich. Nitric acid (≥99.0%), potassium chlorate (≥99.0%), (3-aminopropyl) triethoxysilane (APTES) (≥98.0%), α-bromoisobutyryl bromide (BIBB) (98.0%), triethylamine (≥98.0%), N,N,N′,N″,N″-pentamethyldiethylenetriamine (PMDETA, 99%), ascorbic acid (≥99.0%), decalin (decahydronaphthalene, mixture of cis + trans) (98%), dichloromethane, N,N-dimethylformamide (DMF), sulfuric acid (95–97%) and sodium nitrate were supplied by Sigma-Aldrich. Potassium permanganate and copper (II) bromide were obtained from Merck.

Monomethyl itaconate (MMI) [21], graphene oxide (GO) [16], reduced graphene oxide (rGO) [16], and poly (monomethyl Itaconate) grafted graphene oxide (GO-g-PMMI) [16] was synthesized using the previously reported method.

### 2.2. Synthesis of Hybrid Nanomaterial Based on Graphene Oxide Grafted with PMMI and Barium Titanate Nanoparticles (GO-g-PMMI/BTN)

#### 2.2.1. Hydroxylation of Barium Titanate Nanoparticles

15.250 g of BTN in 80 mL of 30% H2O2 was added to a 2-neck flask. This mixture was sonicated for 30 min (25% amplitude). Next, it was left to react using a refluxing system at 105 °C for 4 h. The resulting suspension was centrifuged and then the obtained solid was washed with three portions of 50 mL distilled water. The solid was dried in a vacuum oven at 80 °C for 12 h. The weight of the white solid was of 14.000 g, and the resulting solid was designated as BTN-OH.

#### 2.2.2. Silanization of Barium Titanate Nanoparticles

In a 250 mL three-necked flask, 2.000 g of BTN-OH and 120 mL of ethanol were added, and the mixing was sonicated for 30 min. Next, the flask was connected to a reflux condenser, a thermometer, and an addition funnel containing a 3 vol.% solution of APTES, which was slowly added drop by drop. Afterwards, the flask was heated at 80 °C, and left to react for 2 h under magnetic stirring. Once the reaction time was elapsed, the reaction mixture was centrifuged and the solid was washed with three portions of 50 mL of distilled water and then dried at 80 °C for 24 h. The weight of the resulting white-beige colored solid was 1.945 g and it was designated as BTN-NH_2_.

#### 2.2.3. Synthesis of the GO-g-PMMI/BTN

In a 250 mL two-necked flask, 0.052 g of GO-g-PMMI, 0.107 g of BTN-NH2, 150 mL of decahydronaphthalene (decalin) and 5 g of 4Å molecular sieve were added. The mixture was conditioned under an inert atmosphere and allowed to react at 160 °C for 4 h. Next, the reaction mixture was filtered under reduced pressure. The solid obtained was washed three times with 50 mL portions of petroleum ether and subsequently three times with 50 mL portions of acetone. Finally, the solid product was dried at 80 °C in a vacuum oven for 12 h, yielding 0.525 g of a gray solid designated as GO-g-PMMI/BTN.

### 2.3. Preparation of Nanocomposites

First, a 18 *w/v*% solution of PEI was prepared by dissolving 0.9 g of PEI in 5 mL of dichloromethane. The mixture was stirred for 30 min, achieving complete dissolution of the polymer. On the other hand, the filler material was weighed and added to a beaker according to the filler percentage stipulated for each nanocomposite and 5 mL of dichloromethane was also added. The masses of the fillers are shown in Table 1.

This mixture was sonicated for 5 min at 25% amplitude to disperse the filler particles and was mixed with the polymer solution under magnetic stirring. The resulting mixture was sonicated for 5 min at 25% amplitude and poured into a Petri dish. The Petri dish was covered with a beaker to favor slow evaporation of the solvent. This procedure was used to obtain all the nanocomposites for evaluation.

### 2.4. Characterization

FTIR spectra were recorded using a Thermo Scientific Nicolet IS50 spectrophotometer (Waltham, MA, USA) with the attenuated total reflectance technique (ATR). The Raman spectra were registered using Raman WITec Alpha 300 RA spectrometer equipped with a 532 nm wavelength laser and 0.2 cm^−1^ resolution. X-ray diffraction analysis was recorded using a Bruker D8 Advance diffractometer (Billerica, MA, USA). The radiation frequency was the Kα line from Cu (1.5406 Å) with a power supply of 40 kV and 40 mA. The morphologies of the samples were obtained by scanning electron microscopy (SEM) and (STEM) using JSM-IT300LV microscope, Jeol (Tokyo, Japan). The samples were coated with an ultra-thin gold (Au) layer. The accelerating voltage was 10 kV. The DC electrical conductivity of samples were determined by using 8.0 cm diameter disc-shaped films using a Keithley High Resistivity Tester model 6517B (Cleveland, OH, USA) and a Resistivity Test Fixture 8009. Broadband dielectric spectroscopic data were obtained using a broadband dielectric spectrometer model BDS-40, Novocontrol Technologies GmbH (Hundsangen, Germany), over a frequency range window of 10^−1^ Hz to 10^6^ Hz and at room temperature. The applied amplitude of the alternating current (A.C.) was 1 V.

## 3. Results and Discussion

**Figure 2:**
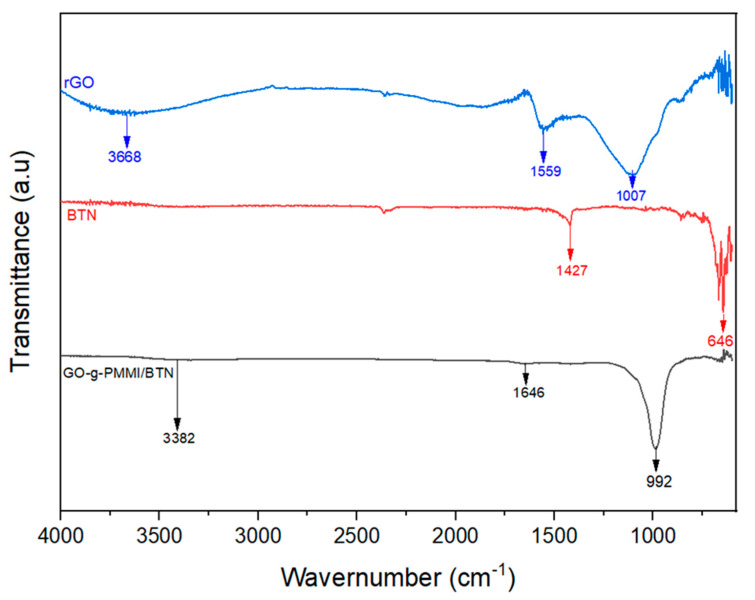
FTIR spectrum of rGO, BTN, and GO-g-PMMI/BTN.

Figure 2 presents the FTIR spectra of reduced graphene oxide (rGO), barium titanate nanoparticles (BTN) and hybrid material based on graphene oxide grafted with poly(monomethyl itaconate) modified with barium titanate (GO-g-PMMI/BTN).

A wide absorption band between 3200 and 3600 in the spectrum of rGO is observed, which is attributed to the presence of hydroxyl groups [22]. Likewise, the absorption band at 1559 cm^−1^ corresponds to the aromatic C=C, which indicates the presence of a conjugated sp^2^ system [23]. Moreover, a band at 1007 cm^−1^ corresponds to hydroxyl and ether groups [24].

On the other hand, the BTN shows only two absorption bands at 1427 cm^−1^ and 646 cm^−1^. The band at 1427 cm^−1^ corresponds to the stretching vibration of $-{\mathrm{CO}}_{3}^{2-}\;$ from residual calcium carbonate from barium titanate synthesis and the band at 646 cm^−1^ represents the Ti-O bond vibration in BaTiO_3_ [25].

In the case of the GO-g-PMMI/BTN spectrum, a band ca. 3382 cm^−1^ is observed, which is attributed to the stretching of the N-H bond associated with the secondary amide yielded by the reaction between BTN-NH_2_ and GO-g-PMMI. The absorption band appearing at 1646 cm^−1^ corresponds to the C=O stretching of the amide groups. Additionally, the band at 992 cm^−1^ was attributed to the Si-O-C group.

**Figure 3:**
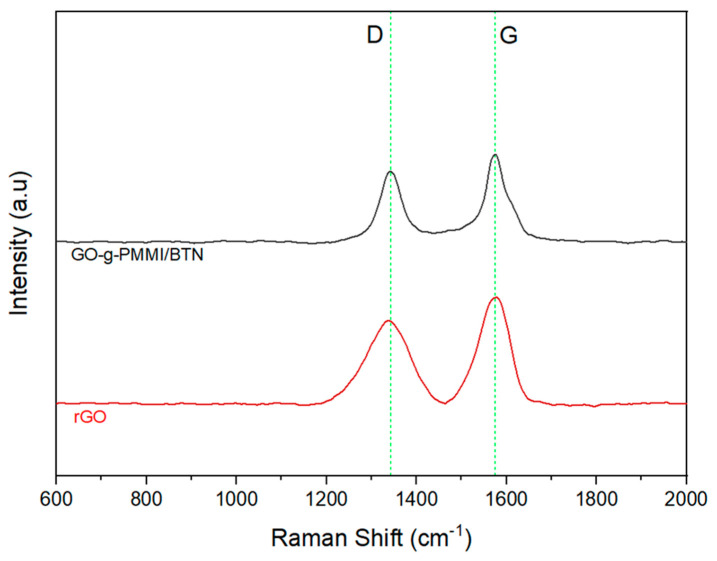
Raman spectrum of rGO and GO-g-PMMI/BTN.

The Raman spectra of rGO and GO-g-PMMI/BTN are depicted in Figure 3. As expected, these materials present characteristic bands, namely D-band and G-band. The D-band is due to the breathing mode of k-point photons of A_1g_ symmetry, and the G-band arises from the first order scattering of E_2g_ phonons with sp_2_ carbon atoms [26]. The intensity of these bands is different, which is attributed to the differences between the graphenic material. Although GO-g-PMMI/BTN presents D and G bands with higher intensity than rGO, the D and G bands appear narrower, and their width differs (see Table 2). Considering the study reported by J. Liu et al. [27], these differences suggest that the charge transport of both graphenic materials will be different.

**Figure 4:**
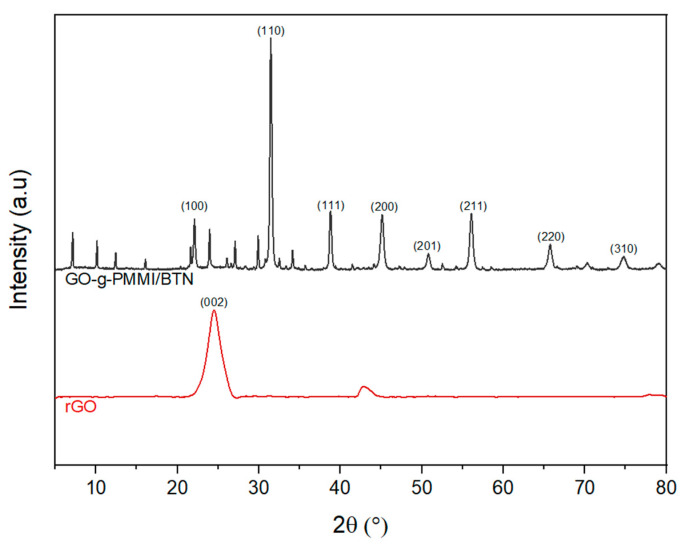
X-ray diffraction patterns of rGO and GO-g-PMMI/BTN.

Figure 4 shows the XRD patterns of rGO and GO-g-PMMI/BTN. The characteristic diffraction peaks at 24.48° and 42.71° correspond to the (002) and (101) characteristic diffraction planes of the graphitic structure of rGO [28], which are randomly rearranged after the graphene oxide (GO) reduction process to rGO. For the GO-g-PMMI/BTN, the characteristic diffraction peaks of BTN appeared at 2θ = 22.05°, 31.42°, 38.86°, 45.18°, 50.80°, 56.15°, 65.64°, and 74.89°, which are associated with the typical structure of a perovskite, such as barium titanate, of (100), (110), (111), (200), (201), (211), (220) and (310) diffraction planes, respectively [29,30].

These X-ray diffraction results suggest that in the process of incorporating barium titanate nanoparticles in GO-g-PMMI, the crystal structure of BTN was not modified.

**Figure 5:**
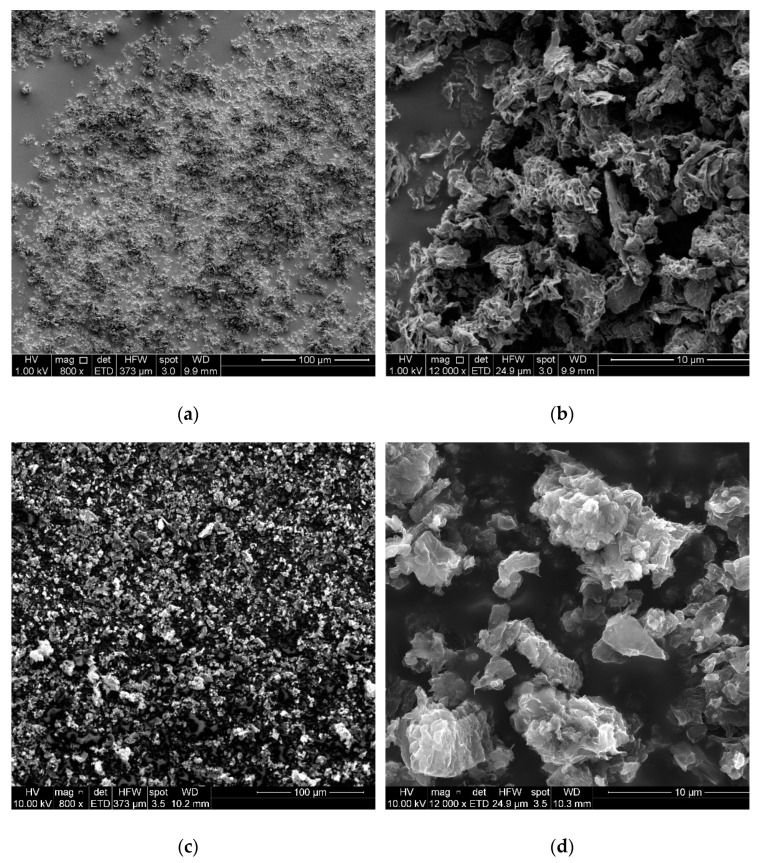
SEM images of (**a**) rGO 800×, (**b**) rGO 12,000×, (**c**) GO-g-PMMI/BTN 800× and (**d**) GO-g-PMMI/BTN 12,000×.

The surface morphology of rGO and GO-g-PMMI/BTN was investigated by using SEM. As seen in Figure 5, there are notable morphological differences between the filler materials.

Figure 5a,b correspond to the rGO showing a compact structure of randomly stacked sheets because of the reduction process to which the graphene oxide was subjected. The rGO morphology appears as an interlinked layered structure [22]. Figure 5c,d correspond to the GO-g-PMMI/BTN. This material has a heterogenous shape, where flakes and particles appeared randomly dispersed.

**Figure 6:**
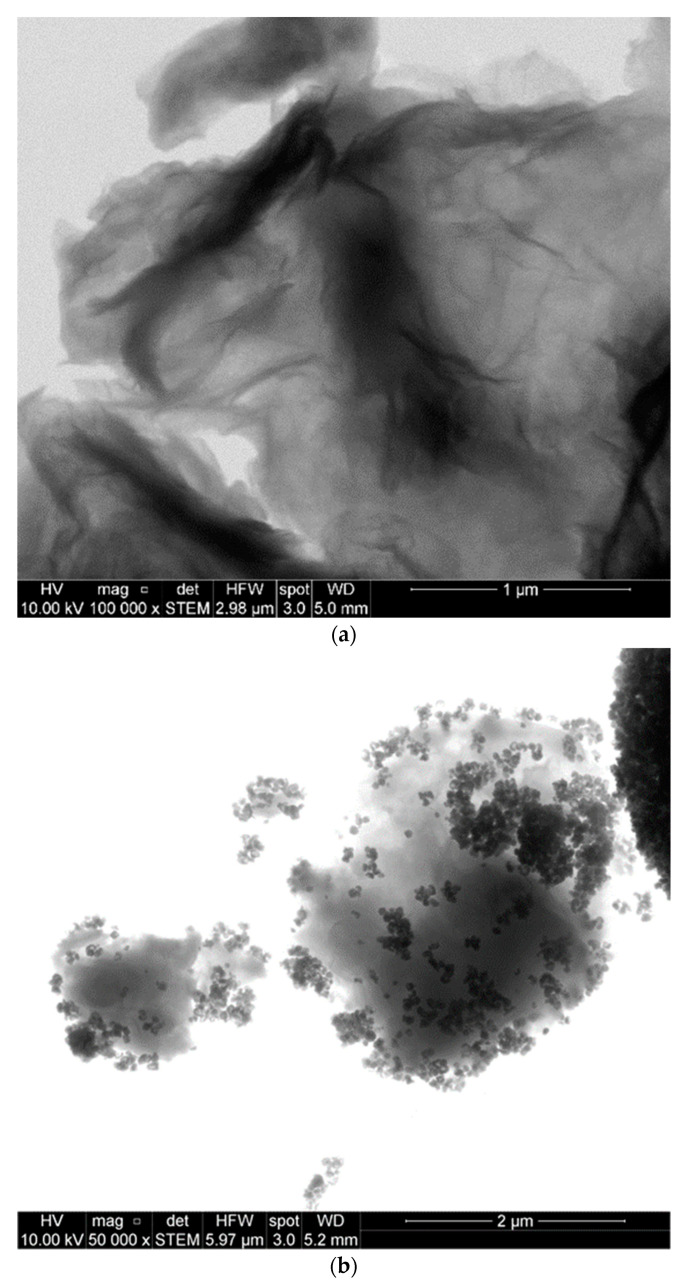
STEM images of rGO (**a**) and GO-g-PMMI/BTN (**b**), both at a magnification of 100,000×.

STEM images of rGO and GO-g-PMMI/BTN are presented in Figure 6. Figure 6a shows the micrograph of rGO at a magnification of 100,000×, where rGO layers are stacked together. The micrograph of GO-g-PMMI/BTN is shown in Figure 5b at a magnification of 100,000×.

**Figure 7:**
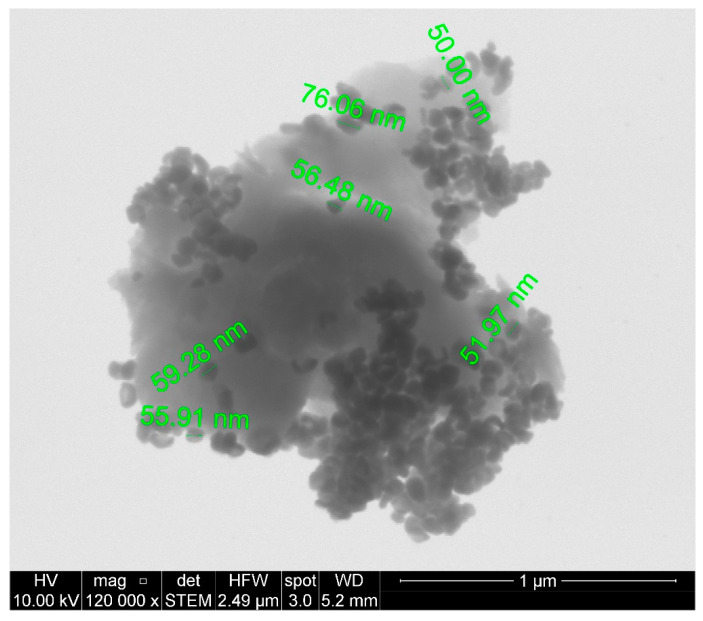
STEM image of GO-g-PMMI/BTN 120,000×, showing the presence of barium titanate nanoparticles on the surface of the GO-g-PMMI.

A dense structure formed by several stacked layers of GO is observed. Moreover, an amorphous structure corresponding to the presence of poly(monomethyl itaconate) on the surface of graphene oxide is observed. The presence of small particles randomly distributed onto the graphene oxide layer in Figure 6b are attributed to the barium titanate nanoparticles. As shown in Figure 7, the sizes of these particles are in the order of 50 to 76 nm. The nanoparticles tend to occupy the edges of the graphene oxide layers, probably due to the oxygen functional groups and, consequently, the brushes of poly(monomethyl itaconate) to which the nanoparticles would be linked [16].

**Figure 8:**
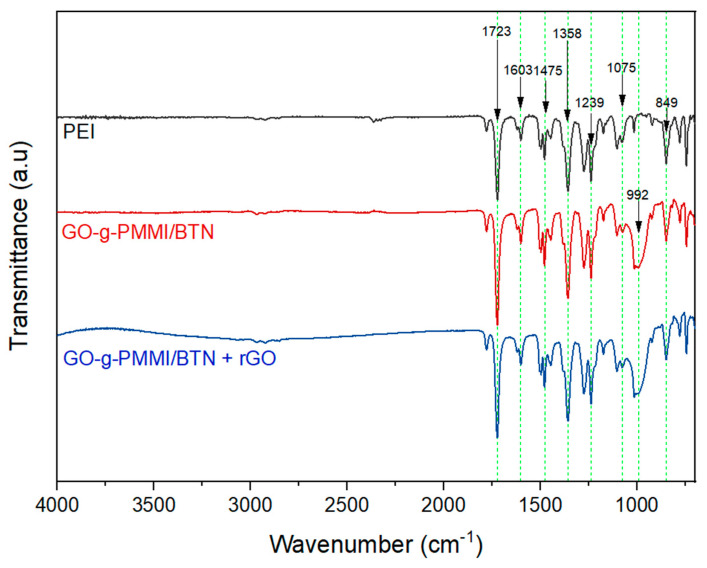
FTIR spectra of GO-g-PMMI/BTN nanocomposite and GO-g-PMMI/BTN + rGO nanocomposite.

The prepared nanocomposites using polyetherimide as the polymeric matrix and rGO and GO-g-PMMI/BTN as filler materials was characterized by FTIR. The results are shown in Figure 8.

The FTIR spectra of PEI show an absorption band at 1723 cm^−1^ typical of imide carbonyl C=O asymmetrical and symmetrical stretching. The absorption bands at 1603 cm^−1^ and 1475 cm^−1^ correspond to the stretching vibration of the C=C aromatic system. The other two absorption bands at 1358 cm^−1^ and 1239 cm^−1^ were assigned to C-N stretching and bending, and to the C-O-C aromatic ether group, respectively [31]. A band at 1075 cm^−1^ was attributed to the tertiary -O-C stretching and the band at 850 cm^−1^ to the of the cyano group (-CN).

The GO-g-PMMI/BTN + rGO nanocomposite shows the absorption bands corresponding to PEI. However, an additional wide band is observed at ca. 992 cm^−1^ attributed to the vibration of the Si-O-C and the alkoxy groups present in the GO-g-PMMI/BTN structure. These facts demonstrate the incorporation of the fillers in the polymer matrix.

**Figure 9:**
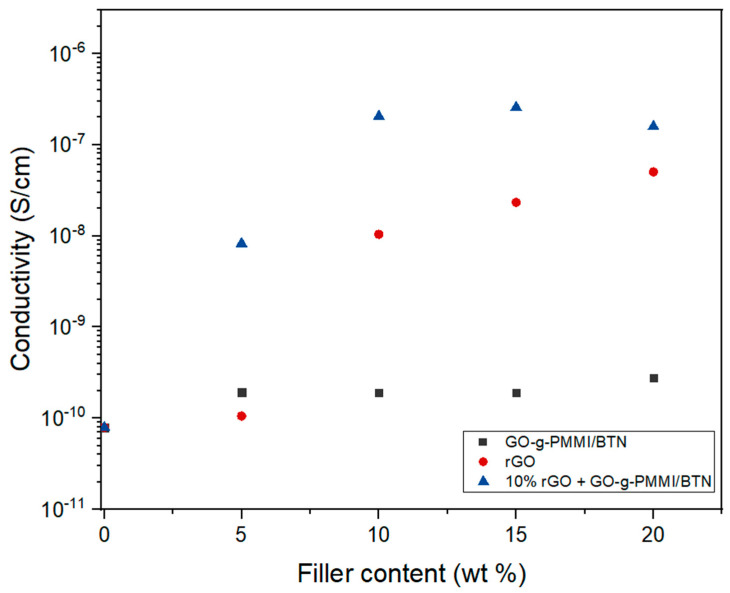
Electrical conductivities of rGO, GO-g-PMMI/BTN and GO-g-PMMI/BTN + 10 wt.% rGO nanocomposites.

The direct current (DC) electrical conductivity of the nanocomposites was evaluated and the results for the nanocomposites filled with 10 wt.% GO-g-PMMI/BTN, 10 wt.% rGO, and 10 wt.% GO-g-PMMI/BTN plus 10 wt.% rGO are shown in Figure 9.

As is known, electrical conductivity, σ, is a measure of the ability of a substance or medium to conduct electricity [32]. The conductivity of the PEI used as polymeric matrix is 1 × 10^−11^ S/cm, however, by incorporating different amounts of GO-g-PMMI/BTN and/or rGO as fillers, the electrical conductivity of the resulting nanocomposites showed variations in respect to that of PEI. The nanocomposite containing a fixed concentration of 10 wt.% rGO and various concentrations of GO-g-PMMI/BTN (5, 10, 15, and 20) wt.% showed the highest conductivity value, being in the order of 1 × 10^−7^ S/cm, followed by the nanocomposite filled with only 10 wt.% rGO, whose conductivity value was in the order of 1 × 10^−8^ S/cm. In the case of the nanocomposite containing 10 wt.% of GO-g-PMMI/BTN, no increase in conductivity was observed, remaining in the order of 1 × 10^−11^ S/cm. From these results, it can be highlighted that GO-g-PMMI/BTN does not improve the conductivity of the nanocomposite. However, GO-g-PMMI/BTN in combination with rGO improves the conductive properties of the nanocomposites above the values obtained for nanocomposites containing rGO as unique filling material (Figure 9). The increase of the electrical conductivity with the increase of the filler content is attributed to a percolation effect imparted by the graphene and hybrid graphene fillers. In fact, the presence of the filler leads to the formation of an electrical percolation network, and the charge transport becomes more efficient with the increase of the conductive filler [3].

**Figure 10:**
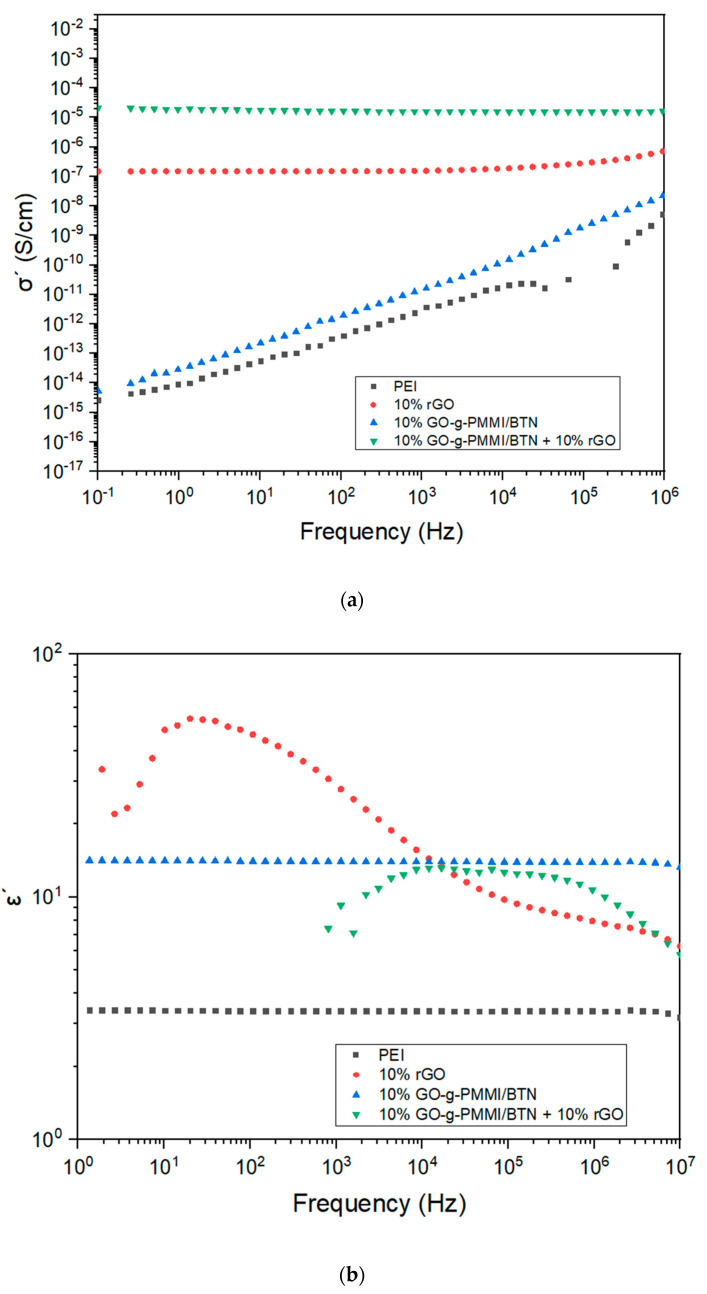
Conductivity (**a**) and dielectric permittivity (**b**) of PEI and nanocomposites filled with rGO, GO-g-PMMI/BTN and GO-g-PMMI/BTN+10% rGO as a function of the frequency.

Figure 10a shows the real part (σ’) of the complex electrical conductivities of PEI and different nanocomposites as functions of the frequency of the electric field. It was observed that the electrical conductivity of PEI increased dramatically by adding 10 wt.% GO-g-PMMI/BTN+10 wt.% rGO (σ’ = 1 × 10^−5^ S/cm, recorded at ν = 10^−1^ Hz) followed by the nanocomposite filled with 10 wt.% rGO (σ’ = 1 × 10^−7^ S/cm, recorded at ν = 10^−1^ Hz), where in both cases the conductivity behavior was independent of the frequency. This indicates that the charge transport is favored by the presence of graphene material [33]. Contrary to the nanocomposite filled only with GO-g-PMMI/BTN, which presented an electrical conductivity close to the conductivity of the polymeric matrix (σ’ = 1 × 10^−15^ S/cm, recorded at ν = 10^−1^ Hz) and the behavior of its conductivity increased with increasing frequency (σ’ = 1 × 10^−8^ S/cm, recorded at ν = 10^6^ Hz). Besides, the discontinuity of the conductivity curve ca. 10x Hz is due to the logarithmic scale applied to the negative values registered. The negative value of the electrical conductivity could be attributed to the electrical current flows against the direction of the electric field [34].

Permittivity is a measure of the electrical polarizability of a material. The charges of a material exposed to an electric field undergo temporary displacement; this generates an induced field within the material [35]. Dielectric permittivity (ε’), which is also known as the relative dielectric constant, is the real part of the complex dielectric permittivity (ε = ε’ − jε”) [36]. Figure 10b presents ε’ as function of the frequency of the applied electric field. It is possible to notice that the nanocomposites filled with 10 wt.% by weight of rGO and 10 wt.% of GO-g-PMMI/BTN + 10 wt.% rGO show a decrease in their ε’ values with increasing frequency. The opposite was the case for the nanocomposite filled with 10 wt.% of GO-g-PMMI/BTN, whose value of ε’ remained constant with the variation of the frequency and showed a dielectric permittivity one order higher than PEI. This indicates the effective contribution of barium titanate to the permittivity of the nanocomposite. It is important to notice that the dielectric curves of the nanocomposites containing hybrid filler and rGO do not present registers below 10^3^ Hz. This is because the curves are represented in a logarithmic scale, and the permittivity values at these frequencies are negative. The negative values of the permittivity are being related with opposed polarization to the electric field. The materials that present negative dielectric constant are considered as revolutionary materials for electronic and photonic applications [37].

**Figure 11:**
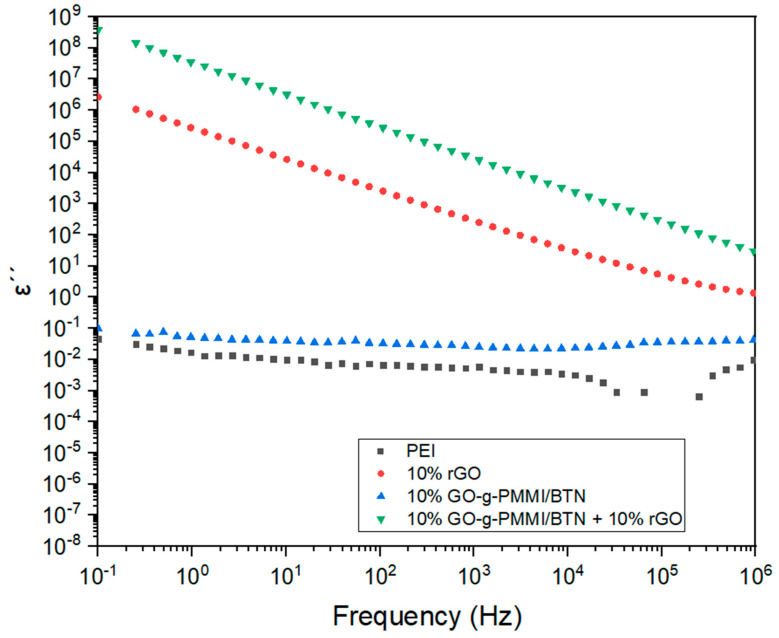
Loss factors of PEI and nanocomposites filled with rGO, GO-g-PMMI/BTN, and GO-g-PMMI/BTN+10 wt.% rGO as a function of frequency.

Figure 11 shows the results obtained of the dielectric loss (ε’’) for PEI and the nanocomposites filled with rGO, GO-g-PMMI/BTN, or GO-g-PMMI/BTN+10 wt.% rGO as a function of the frequency of the applied electric field in the range between 10^−1^ and 10^6^ Hz, observing a gradual decrease for GO-g-PMMI-filled nanocomposites (ε’’ = 3.818 × 10^8^, recorded at v = 10^−1^ to ε’’ = 3.053 × 10^1^, recorded at v = 10^−6^), as well as for those filled with rGO (ε’’ = 2.666 × 10^6^ S/cm, recorded at v = 10^−1^ to ε’’ = 1.35 S/cm, recorded at v = 10^−6^). The incorporation of 10 wt.% of rGO to the nanocomposite containing GO-g-PMMI/BTN improved the dielectric permittivity of the material since the nanocomposites with GO-g-PMMI/BTN as filler presented a lower dielectric permittivity (ε’’ = 0.097, recorded at ν = 10^−1^ Hz) whose value did not vary with the increase in frequency.

## 4. Conclusions

Nanocomposites based on polyetherimides filled with rGO, GO-g-PMMI/BTN or GO-g-PMMI/BTN+10 wt.% rGO, a graphene oxide hybrid material obtained from GO grafted with poly(monomethyl itaconate) modified with barium titanate nanoparticles, were prepared and their electrical properties were studied. It was observed that the electrical properties of the nanocomposites varied depending on the type of the filler material used. The nanocomposites filled with 10wt.% of GO-g-PMMI/BTN presented the same conductivity of 1 × 10^−11^ S/cm shown by the polymeric matrix. On the other hand, the nanocomposites filled with 10wt.% of rGO presented a conductivity in the order of 1 × 10^−8^ S/cm. The incorporation of 10 wt.% rGO with 10 wt.% GO-g-PMMI/BTN as fillers in PEI allowed us to obtain a new nanocomposite material with improved electrical properties compared with the nanocomposite containing only 10wt.% rGO. The conductivity of this new nanocomposite was in the order of 1 × 10^−7^ S/cm. The combination of rGO and GO-g-PMMI/BTN hybrid material improved the electrical conductivity of the resulting nanocomposite.

## Figures and Tables

**Table 1:** Mass of PEI and fillers used for the preparation of the nanocomposites.

Compound	PEI (g)	GO-g-PMMI/BTN (g)	rGO (g)
PEI	0.9000	0	0
GO-g-PMMI/BTN	0.9200	0.0937	0
rGO	0.9300	0	0.0936
GO-g-PMMI/BTN + rGO	0.9300	0.0946	0.0937

**Table 2:** Values of I_D_/I_G_, FWHM and the Raman shifts of the D-band and G-band.

Sample	Raman Shift (cm^−1^)		FWHM		I_D_/I_G_
	D	G	D	G	
rGO	1335	1576	58.898	83.643	0.837
GO-g-PMMI/BTN	1343	1574	51.320	54.610	0.962

## Data Availability

Data sharing not applicable.
